# Pharmacovigilance Practices for Better Healthcare Delivery: Knowledge and Attitude Study in the National Malaria Control Programme of India

**DOI:** 10.1155/2014/837427

**Published:** 2014-09-15

**Authors:** Pooja Gupta, Anupkumar R. Anvikar, Neena Valecha, Yogendra K. Gupta

**Affiliations:** ^1^Department of Pharmacology, All India Institute of Medical Sciences, New Delhi 110029, India; ^2^National Institute of Malaria Research, Indian Council of Medical Research, New Delhi 110077, India; ^3^Pharmacovigilance Programme of India, India

## Abstract

*Objective.* With large scale rollout of artemisinin based therapy in the National Malaria Control Programme of India, a risk management plan is needed. This depends on adverse drug reaction (ADR) reporting by the healthcare professionals (HCPs). For the programme to be successful, an understanding of the mindset of HCPs is critical. Hence, the present study was designed to assess and compare the ADR reporting beliefs of HCPs involved in the National Malaria Control Programme of India. *Methods.* A cross–sectional survey was conducted amongst the HCPs who manage malaria up to the district level in India. A 5-point Likert scale-based questionnaire was developed as a study tool. *Results.* A total of 154 HCPs participated in the study (age: 42.4 ± 10.1 years with 33.8% being females). About 61% felt that only medically qualified HCPs are responsible for ADR reporting. Likeliness to report in future was mentioned by 45% HCPs. The knowledge score was relatively lower for life science graduates (*P* = 0.09). Knowledge correlated positively with attitude (*r* ^2^ = 0.114; *P* < 0.0001). *Conclusion.* Based on the caveats identified, a specific and targeted in-service education with hands-on training on ADR monitoring and reporting needs to be designed to boost real time pharmacovigilance in India.

## 1. Introduction

Antimalarials are frequently used for presumptive treatment of fever especially in malaria endemic regions [1]. In view of the increasing resistance to existing antimalarials, India switched to artemisinin based therapy as first line antimalarial treatment in its National Vector Borne Disease Control Programme. In 2005, artemisinin based therapy was introduced in India in the form of artesunate plus sulfadoxine and pyrimethamine in areas with chloroquine failure and later extended to all falciparum malaria cases across the country in 2010. These drugs are highly effective and their free distribution through the national programme may increase the risk of adverse drug reactions (ADRs) and drug resistance. Hence, the recent change in prescribing practice necessitates pharmacovigilance of antimalarial drugs especially artemisinin based therapy.

The World Health Organization defines pharmacovigilance as “the science and activities relating to the detection, assessment, understanding and prevention of adverse effects or any other drug-related problem.” The adverse drug reaction reporting has been going on in India for a long time. The current Pharmacovigilance Programme of India started in 2010 and has organized nearly 30 workshops on pharmacovigilance in the last two years. More than 32,000 ADR reports have been collected through ADR monitoring centres housed in 60 medical colleges across the country. However, pharmacovigilance has been associated with a national health programme for the first time in the country. ADR reporting barely existed for antimalarial drugs before the start of this project. The objectives are to monitor ADRs to antimalarial medicines and guide pharmacovigilance capacity building in the country through cohort event monitoring for antimalarial drugs.

Good pharmacovigilance practices based on knowledge and attitude are the key to safeguard against seven individual behavioral variations and societal barriers which lead to underreporting of ADRs [2, 3]. Studies on knowledge and attitude of healthcare professionals (HCPs) have shown high variation. In India, such data is limited to tertiary care hospitals [4–6]. However, 69% of Indian population lives as agrarian society in nearly 6.4 Lakh rural units [7]. Thus, the present study aimed at assessing and comparing the knowledge and attitude of healthcare professionals with medical and other life sciences background, involved in National malaria control programme in India.

## 2. Methods

The study was conducted as a cross-sectional, voluntary, and anonymized survey between May 2010 and April 2012. It was carried out as a part of cohort event monitoring of antimalarial drugs. The survey was administered to the participants prior to the pharmacovigilance awareness and training workshops. The states with an annual parasite incidence more than 2 were considered for the workshop. Of these, four states in different regions (Gujarat, Madhya Pradesh, Assam, and Karnataka) were selected for conducting awareness workshops. HCPs from Arunachal Pradesh were invited for the Assam workshop for logistic reasons. The survey population comprised of HCPs involved in malaria management at the level of (a) peripheral rural/urban health centre, (b) community health centre, or (c) district health centre. The HCPs listed with the regional medical research centre of the participating state were invited for the workshop. Of the HCPs participating in the workshop, all those willing to contribute to the survey were selected. The questionnaire was administered to the participants by the paramedical staff of regional medical research centres. The participants were allowed to question the investigators to get their doubts cleared. The study was approved by the Institute Ethics Committee, All India Institute of Medical Sciences, New Delhi, India.

A short, easy-to-use, self-administered questionnaire was designed to capture (i) demographic characteristics including age, gender, qualification, length of experience in malaria control programme, and previous training in adverse event monitoring and (ii) 21 items assessing subjects' knowledge and attitude regarding adverse event reporting and pharmacovigilance (Table 1). Each statement had a corresponding 5-point Likert rating scale (1 = strongly agree to 5 = strongly disagree). A combination of positive and negative statements was used and scores were reversed where appropriate. The questionnaire was pretested in two different language speaking states, that is, Gujarat and Karnataka, in 20 subjects to assess reproducibility and suitability. The pretest participants were then contacted for their feedback and understanding with respect to the questionnaire.

Statistical analysis was carried out using Stata (College station, Texas, USA; version 11.0). Items measuring similar concept were grouped together as either knowledge items or attitude items. Separate scores were calculated for both by summing individual item scores. Items which were not attempted were treated as missing. The Cronbach alpha of 0.7 for the questionnaire suggests good internal consistency and an overall reliability. One-way ANOVA with Bonferroni post hoc analysis, Student's *t*-test, and Pearson correlation coefficient were used, as appropriate. A *P* < 0.05 was considered significant.

## 3. Results

### 3.1. Demographics of Responses

A total of 250 questionnaires were administered of which 154 (61.6%) were returned completely filled. The 154 participating healthcare professionals, of whom 33.8% were females, were from 38 districts of 5 states. Mean age of the participants was 42.4 years (range: 26–62 years). The participants reported having an experience in malaria management for 11.9 years (range: 1–37 years). There were 25% medical postgraduates, 37% medical graduates, 18% life science postgraduates, and 20% life science graduates. Fifty-seven participants reported having observed at least one ADR and thirty-five responded as having reported an ADR.

### 3.2. Knowledge

Awareness regarding the Pharmacovigilance Programme of India was informed by 50% reporters. While 61% thought that only medically qualified doctors were responsible for ADR reporting, eight (5%) participants were aware of the fact that all healthcare professionals should report ADRs. Participants (76%) considered that ADRs due to antimalarials should be reported. Vaccines and antibiotics were considered as reportable by 67% and 54% participants, respectively; ten (6.5%) participants correctly identified all the listed reportable therapeutic options. All the listed benefits of ADR reporting were accurately recognized by 19 (12%) reporters.

ADR reporting was accepted to improve patient safety by 98% participants. Less than half the participants (35%) knew that all ADRs are not known when a new drug comes to the market. Participants (65%) further thought that they needed to be sure of causality to report ADRs. Results for other knowledge items are summarized in Table 2. The total score for knowledge was computed as sum of individual item scores with a possible range of 6–48. The mean total score for knowledge was 25.5 ± 5.1 (range: 12–41).

### 3.3. Attitude

Significance of reporting even a single ADR was felt by 60% participants but likeliness to report in future was suggested by 45%. Lack of time and fear of problems for self were reported to be important deterrents by 10% subjects. Other possible deterrents for reporting are listed in Table 2. Monetary benefits for reporting were opted for by 27.9% subjects. Reporting was also considered as blame on the reporting HCP for patient harm by 22.7%. Respondents (24.7%) felt that ADR reporting was not part of their job and another 14.3% were not sure. There was divided opinion on whether reporting should be voluntary (Table 2). The total score for attitude was computed as sum of individual item scores with a possible range of 11–55. The mean total score for attitude was 42.1 ± 5.97 (range: 28–54).

### 3.4. Attributes Affecting Knowledge and Attitude

**Figure 1:**
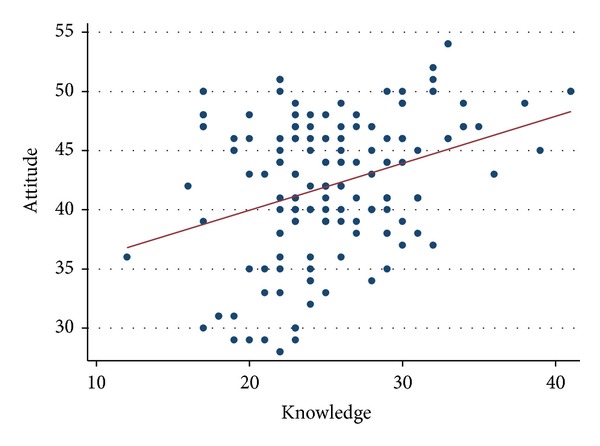
Scatter plot demonstrating the relationship between total knowledge and attitude score towards ADR reporting in healthcare professionals working at peripheral health centres in India.

Knowledge correlated negatively with age (*r* = −0.16; *P* = 0.05) but was not associated with gender (*P* = 0.09). The knowledge score for life science graduates and postgraduates was 22.4 ± 4.3 and 24.9 ± 6.3, respectively. For medical graduates and postgraduates, the score was 26.5 ± 4.4 and 26.7 ± 4.7, respectively. However the overall difference was not statistically significant (*P* = 0.09). The effect of a previous training in ADR reporting on knowledge was not significant (*P* = 0.16). Of the 139 participants who responded to this question, 84% had not received any training. No significant difference in attitude was observed based on age, gender, qualification, or prior training in ADR reporting. The percentage score for knowledge was 62 ± 12% versus 78 ± 11% for attitude.  Figure 1 shows the correlation between knowledge and attitude (*r* = 0.34; *P* < 0.0001).

## 4. Discussion

Pharmacovigilance helps in optimizing the management of resources, especially in the national health programmes of developing countries [8]. Due to recent, large scale introduction of artemisinin combination therapy in National Malaria Control Programme of India, cohort event monitoring was started. Knowledge is considered a prerequisite and attitude a determinant of ultimate ADR reporting [9]. A good knowledge of pharmacovigilance and positive attitude towards reporting at the peripheral level would assist in better implementation of pharmacovigilance for antimalarial drugs. Hence, our study aimed at identifying the gaps in knowledge and attitude of Indian healthcare professionals involved in management of malaria at the peripheral level.

There was a nearly uniform representation from healthcare professionals with graduate and postgraduate degrees in medical and other life sciences. The participants belonged to a wide age range and accordingly had a varied duration of experience in malaria management. Age of the reporters was observed to correlate inversely with the total knowledge score. Previous studies have reported either no effect or a direct relationship between age and knowledge [10–12]. In our study, although the inverse relationship was not significant, it could be due to the recent changes like increasing introduction of pharmacovigilance in the undergraduate and postgraduate curricula, establishment of a pharmacovigilance centre in medical colleges recognized by Medical Council of India, and conduct of awareness programmes in the country [13]. Despite a higher knowledge score for younger participants, their attitude was similar to the older ones. It could possibly be due to an indifference towards the importance of individual contribution. Gender had no effect on knowledge of attitude regarding pharmacovigilance, in line with previous Swedish and Malaysian studies [10, 14]. However, a study conducted in Nepal reported significantly better questionnaire score in males [12].

Approximately half the participants were aware of the existence of Pharmacovigilance Programme of India. As may be expected, the awareness was less than the 73% reported for a tertiary care hospital in Delhi [15]. In Germany, 80% participants in a study were aware of their national pharmacovigilance programme [16]. This suggests the need for continued intervention amongst HCPs to promote ADR reporting. We observed a lack of knowledge in our study. Many participants were not aware who should report ADRs, what type of medicinal products should monitored for ADRs and what are the benefits of pharmacovigilance. Only 15% participants believed that ADRs with complementary and alternative medicine should be reported. This is in contrast to a Malaysian study where 69% pharmacists believed in reporting these ADRs [14]. Clarification is needed on who should report, what to report, where to report, and how to report.

A large proportion of professionals in the study believe that all ADRs are known when a drug is commercialized which is in line with a Chinese study [17]. More than one-third of the participants were either neutral or did not consider ADR reporting as a professional obligation and felt that they should be remunerated for reporting. This is in contrast to a Spanish study in which ADR reporting was considered a part of job description for which no incentive was required [18]. ADR reporting is often perceived as a time consuming activity [19–21]. However, our study did not note time as a critical obstruction towards ADR reporting, in line with a Dutch study [9]. The opinion on voluntary spontaneous reporting was also divided in our study. The results of our study suggest factors like the need to establish causality, need for incentive, and mixed opinion on voluntariness of ADR reporting system as strong deterrents to ADR reporting. The possible reasons could be patient overload, excessive paper work, and lack of infrastructure support. However, such mindset could grievously harm ADR reporting for antimalarial drugs as malaria endemic countries frequently suffer from these conditions.

Some of the Inman's “seven deadly sins” presented themselves in our study [22]. A reduction in ignorance, indifference, and complacency has been reported to improve the probability of reporting [23]. A positive relationship between knowledge and attitude and a relatively higher score for attitude suggests the presence of a huge reporting potential in the country. As has been reported previously, practitioners working in primary care were much more likely to report than those working in hospitals [23].

It is important for each country to assess the knowledge and attitude of its HCPs across different healthcare sectors, diseases conditions, and time [24]. This will allow a transformation from one-shoe-fits-all to an evidence-based strategian approach and strengthen the architecture of pharmacovigilance. Harnessing the potential interest of Indian medical fraternity in ADR reporting requires supplementing the knowledge of reporting system and procedures at the smallest units of healthcare in the country.

The results of the study provide baseline data to design training modules for these potential reporters in the country. Educational tools also need to address the legal and social concerns of reporters as advocated by some of the participants. The results will also be used for development of attitude-based interventions in the country. Personal encouragement, recognition, and feedback were identified as driving factors to stimulate reporting. Introducing ADR reporting as an agenda for monthly malaria control meetings will act as reminder at regular intervals. A multipronged approach with thoughtful improvisation will improve the willingness to observe and communicate adverse drug reactions and therefore foster a much needed healthy reporting environment at the peripheral healthcare levels of the country.

## Figures and Tables

**Table 1:** Dimension-wise overview of the items included in the questionnaire.

Knowledge-related items	
All ADRs due to a drug are already known when it first comes to the market∗.	
All ADRs should be reported for newly marketed drugs∗.	
Serious reactions should be reported for old drugs in use for a long time∗.	
I do not need to report minor ADR∗.	
I should report only uncommon ADR∗.	
I should report ADR only when I am sure that it is due to a drug∗.	
Who is qualified to report ADRs (medical doctors, nurses, pharmacists, physiotherapist, or multipurpose healthcare workers)^&^?	
What should be monitored for ADRs (vaccines, complementary medicines, over-the-counter drugs, antibiotics, antimalarials, or topical drugs)^&^?	
Purpose of ADR monitoring is (identifying safe drugs, calculating ADR incidence, identifying predisposing factors, identifying previously unrecognized ADRs, and serving as a source of information, for comparison of drugs within the same therapeutic class)^&.^	
Are you aware of the adverse event monitoring system in India^#^?	

Attitude-related items	

I am likely to report an ADR in future∗.	
ADR reporting improves patient safety∗.	
I am not doing my job properly unless I report ADR∗.	
Only one ADR will not make a significant contribution∗.	
Reporting ADR might create problems for me∗.	
There should be payment for ADR reporting∗.	
Reporting ADR will make me responsible for patient harm∗.	
ADRs are not preventable so there is no point in reporting∗.	
ADR reporting should be voluntary∗.	
I am unlikely to report ADRs due to lack of time∗.	
I do not feel the need to report an ADR that I have recognized∗.	

Demographic characteristics	

Age, gender, qualification, years of experience in malaria control programme, and training in ADR reporting	

*Answered on a 5-point Likert scale; ^&^Answered as multiple correct answer question with one point for each correct answer; ^#^Answered as yes/no.

**Table 2:** Statement-wise responses to knowledge and attitude questionnaire of healthcare professionals on a 5-point Likert scale expressed as absolute numbers (%).

Statement	Strongly disagree	Disagree	Neutral	Agree	Strongly agree
All ADRs are known	21 (13.6)	33 (21.4)	10 (6.5)	60 (39)	30 (19.5)
Report all ADRs for new drugs	6 (3.9)	8 (5.2)	2 (1.3)	36 (23.4)	102 (66.2)
Report only serious ADRs for old drugs	12 (7.8)	8 (5.2)	8 (5.2)	34 (22.1)	92 (59.7)
No need to report minor ADRs	60 (39)	37 (24)	14 (9.1)	28 (18.2)	15 (9.7)
Report only uncommon ADRs	54 (35.1)	32 (20.8)	11 (7.1)	29 (18.8)	28 (18.2)
I should report only when I am sure	20 (13)	18 (11.6)	16 (10.4)	40 (26)	60 (39)
Likely to report future ADRs	15 (11.4)	7 (5.3)	51 (38.6)	53 (40.1)	6 (4.6)
ADR reporting improves safety	1 (0.6)	1 (0.6)	1 (0.6)	21 (13.7)	130 (84.5)
I am not doing my job properly	17 (11)	21 (13.6)	22 (14.3)	42 (27.3)	52 (33.8)
Only one ADR is not significant	62 (40.3)	28 (18.2)	6 (3.9)	37 (24)	21 (13.6)
I fear problems for me	94 (61.1)	29 (18.8)	15 (9.7)	10 (6.5)	6 (3.9)
There should be payment	64 (41.6)	22 (14.3)	25 (16.2)	23 (14.9)	20 (13)
Responsibility for patient harm	83 (53.9)	26 (16.9)	10 (6.5)	13 (8.4)	22 (14.3)
ADR not preventable	103 (66.9)	35 (22.7)	8 (5.2)	7 (4.6)	1 (0.6)
Reporting should be voluntary	41 (26.6)	25 (16.2)	13 (8.5)	40 (26)	35 (22.7)
Unlikely to report for lack of time	86 (55.8)	36 (23.4)	14 (9.1)	13 (8.4)	5 (3.3)
Self-recognized ADRs not reported	87 (56.9)	33 (21.6)	10 (6.5)	14 (9.1)	9 (5.9)
